# Corynebacterium ulcerans Diphtheria in Japan

**DOI:** 10.3201/eid0906.020645

**Published:** 2003-06

**Authors:** Akio Hatanaka, Atsunobu Tsunoda, Makoto Okamoto, Kenji Ooe, Akira Nakamura, Masashi Miyakoshi, Takako Komiya, Motohide Takahashi

**Affiliations:** * Asahi General Hospital, Chiba, Japan; † National Institute of Infectious Diseases, Chiba, Japan

**Keywords:** cat, Corynebacterium ulcerans, diphtheria toxin, dyspnea, erythromycin, Japan, dispatch

**To the Editor**: *Corynebacterium ulcerans* causes a zoonotic infection similar to diphtheria, which is caused by *C. diphtheriae*. Studies indicate that signs and symptoms of a diphtheria-like illness caused by *C. ulcerans* are milder than those caused by *C. diphtheriae*. However, some strains of *C. ulcerans* produce potent diphtheria toxin and may cause severe symptoms similar to those caused by *C. diphtheriae* (*1*). We report a case of a diphtheria-like illness caused by *C. ulcerans* infection.

A previously healthy 52-year-old woman first noticed hoarseness approximately 3 days before admission to the hospital. On February 16, 2001, severe dyspnea and fever developed, and the patient was referred to the emergency room of the Asahi General Hospital by her private practitioner. Physical examination indicated a large stridor, which could be heard without using a stethoscope. Cyanosis was not observed. The endoscopic examination showed a thick white coat covering the nasopharynx and laryngeal vestibulum, and subglottic constriction was also observed. A chest x-ray showed diffuse infiltrates in both lungs. Pertinent laboratory findings on admission included leukocyte count of 16.8 x 10^3^/μL and C-reactive protein of 20.0 mg/dL. The serum level of liver transaminase was normal, and both Wassermann reaction and anti-HIV antibody tests were negative. Pharyngolaryngitis and pneumonia was diagnosed in the patient. Because of severe dyspnea, intubation was performed, which caused sudden and unexpected exacerbation of the condition. Severe cyanosis subsequently developed. Extubation was immediately performed, and a thick white material was found to be filling the lumen of the endotracheal tube. Reintubation was performed, and dyspnea subsided. The patient was hospitalized in the intensive-care unit. Sulbactam sodium/ampicillin sodium (6 g per day) was intravenously administered for 4 days; however, the symptoms were not much improved. The symptoms were most consistent with those of diphtheria. Therefore, the patient was subsequently placed on erythromycin (1.0 g/day) and quickly responded to this treatment without administration of diphtheria antiserum. Erythromycin was intravenously administered at 1 g per day for 9 days, then orally administered at 1,200 mg per day for the next 14 days. Throughout the hospitalization, no complication occurred, and no abnormalities were noted in the electrocardiograms or in the patient’s neurologic status. The patient was discharged uneventfully, and no serious sequelae were noted for 20 months. History of immunization for diphtheria was not known.

After the hospitalization for this acute illness, a laboratory report showed that *C. ulcerans* was cultured from the thick white coat of the throat. No other bacteria were found. The National Institute of Infectious Diseases in Tokyo later confirmed identification of the bacteria. By using Elek’s test, Vero cell toxicity, and polymerase chain reaction for toxigene, this strain of *C. ulcerans* was proven to produce diphtheria toxin identical to *C.* *diphtheriae* (*2*–*4*). Although administering appropriate antibiotics as well as antitoxin is a standard of care for patients with diphtheria, antitoxin was not given to this patient because of her rapid response to the erythromycin therapy.

*C. ulcerans* infections in humans occur after drinking unpasteurized milk or coming in contact with dairy animals or their waste (*5*,*6*). However, person-to-person transmission of *C. ulcerans* has not been reported, and in some cases, the route of transmission is not clear (*7*). Recently, *C. ulcerans*-producing diphtheria toxin was isolated in the United Kingdom from cats with nasal discharge (*8*).

Our patient did not have direct contact with dairy livestock or unpasteurized dairy products; however, more than 10 dairy farms are scattered around her home. Moreover, she kept nearly 20 cats in her house and had been scratched by a stray cat a week before illness developed. This stray cat, which had rhinorrhea and sneezing, had wandered into her house. The stray cat died before the patient became ill, and no further investigation could be made. Stray cats might well be one of the possible carriers of *C. ulcerans* and might have transmitted the bacteria to this patient. To our knowledge, a case of human infection caused by *C. ulcerans* has never been reported in Japan. On the basis of current experience, this bacterium does exist in Japan and can potentially cause a serious diphtheria-like illness in humans.
